# Bilirubin as an Anti-oxidant for Surgical Stress: A Preliminary Report of Bilirubin Oxidative Metabolites

**DOI:** 10.1155/1999/16374

**Published:** 1999-07

**Authors:** N. Kozaki, S. Shimizu, K. Chijiiwa, K. Yamaguchi, S. Kuroki, K. Shimoharada, T. Yamaguchi, H. Nakajima, M. Tanaka

**Affiliations:** 1 First Department of Surgery Kyushu University Faculty of Medicine 3-1-1 Maidashi Fukuoka Higashi-ku 812-8582 Japan; 2 Division of Biochemical Examination Kyushu University Faculty of Medicine Fukuoka Japan; 3 Department of Biochemical Genetics Medical Research Institute Tokyo Medical and Dental University Tokyo Japan

## Abstract

Background Bilirubin has been recognized as an antioxidant. The purpose of this study was to examine whether bilirubin would act as an antioxidant for surgical stress in humans. Materials and Methods Serum bilirubin and urinary bilirubin oxidative metabolites (BOM) were measured in 96 patients who underwent surgery. The antioxidant activity of bilirubin was assessed using BOM measured by enzyme-linked immunosorbent assay with an anti-bilirubin monoclonal antibody. Results Serum bilirubin levels increased after surgery in all 96 patients (*p*<0.01), but did not correlate with operation time or blood loss (*p*=0.53 and *p*=0.28, respectively). BOM increased only in patients with major surgeries (*p*=0.048). Significant correlations between BOM and operation time and blood loss were found (*p*<0.01). Conclusions Bilirubin appears to act as an antioxidant for invasive surgery in humans. Urinary BOM could be a reliable marker for the degree of surgical stress.


*HPB Surgery*, 1999, Vol. 11, pp. 241-248
Reprints available directly from the publisher
Photocopying permitted by license only

© 1999 OPA (Overseas Publishers Association) N.V.
Published by license under
the Harwood Academic Publishers imprint,
part of The Gordon and Breach Publishing Group.
Printed in Malaysia.

### Bilirubin as an Anti-oxidant for Surgical Stress: A Preliminary Report of Bilirubin Oxidative Metabolites

N. KOZAKI[a], S. SHIMIZU[a,*], K. CHIJIIWA[a], K. YAMAGUCHI[a], S. KUROKI[a], K. SHIMOHARADA[b], T. YAMAGUCHI[c], H. NAKAJIMA[c] and M. TANAKA[a]

[a]*First Department of Surgery, Kyushu University Faculty of Medicine, 3-1-1 Maidashi, Higashi-ku, Fukuoka 812-8582, Japan;*
[b]*Division of Biochemical Examination, Kyushu University Faculty of Medicine, Fukuoka Japan;*
[c]*Department of Biochemical Genetics, Medical Research Institute, Tokyo Medical and Dental University, Tokyo, Japan*

*(Received 30 September 1997; In final form 5 February 1998)*

**Background Bilirubin has been recognized as an antioxidant. The purpose of this study was to examine whether bilirubin would act as an antioxidant for surgical stress in humans. Materials and Methods Serum bilirubin and urinary bilirubin oxidative metabolites (BOM) were measured in 96 patients who underwent surgery. The antioxidant activity of bilirubin was assessed using BOM measured by enzyme-linked immunosorbent assay with an anti-bilirubin monoclonal antibody. Results Serum bilirubin levels increased after surgery in all 96 patients ($p < 0.01$), but did not correlate with operation time or blood loss ($p = 0.53$ and $p = 0.28$, respectively). BOM increased only in patients with major surgeries ($p = 0.048$). Significant correlations between BOM and operation time and blood loss were found ($p < 0.01$). Conclusions Bilirubin appears to act as an antioxidant for invasive surgery in humans. Urinary BOM could be a reliable marker for the degree of surgical stress.**

*Keywords:* Bilirubin, antioxidant, surgical stress

### INTRODUCTION

Bilirubin is believed to be cytotoxic, and its toxicity has been reported to be related to injury to the mitochondrial membrane [1] or to immune suppression [2,3]. Operative procedures in patients with jaundice result in more complications and increased mortality in comparison to those without jaundice. Postoperative jaundice is often associated with sepsis or liver failure [4]. Prolonged hyperbilirubinemia has thus been considered to be a dangerous condition both before and after surgery. However, in contrast to the toxic effect, Stocker *et al.*, have demonstrated a beneficial role for bilirubin in that it can act as a powerful antioxidant *in vitro* [5]. This observation was confirmed in rats injected with endotoxin as a source of oxidative stress *in vivo* [6]. Bilirubin oxidative metabolites (BOM) have been identified in human urine. The antioxidant activity of bilirubin can be assessed by measuring these compounds with an enzyme-linked immunosorbent assay (ELISA) [7].

Surgery is one of the oxidative stresses. Many cytokines are released from macrophages, fibroblasts and endothelial cells, during surgery. Neutrophils then increase in number and are

*Corresponding author. Tel.: +81-92-642-5442, Fax: +81-92-642-5458, e-mail: shimizu@surgl.med.kyushu-u.a.c.jp activated to release granular enzymes as well as reactive oxygen species [8,9]. Our current working hypothesis is, therefore, that bilirubin production is accelerated during surgery as a spontaneous protection against oxidative stress. In the present study, we explored the possibility that bilirubin would act as an antioxidant in humans during the perioperative period by measuring urinary BOM as well as serum bilirubin in relation to the severity of surgical injury.

TABLE I Diagnoses of 96 patients

| Diagnosis | Number of patients |
|---|---|
| Non-abdominal diseases | **20** |
| Breast tumor | 7 |
| Thyroid tumor | 6 |
| Inguinal hernia | 3 |
| Anal fistula | 2 |
| Myasthenia gravis | 2 |
| Abdominal diseases | **76** |
| Cholelithiasis | 21 |
| Gastric tumor | 18 |
| Colorectal tumor | 8 |
| Inflammatory intestinal disease | 6 |
| Pancreatic tumor | 5 |
| Other abdominal diseases | 18 |
| Total | **96** |

### MATERIALS AND METHODS

### Patients

Ninty-six patients who underwent surgery from September 1995 to January 1996 in the First Department of Surgery, Kyushu University Hospital, Fukuoka, Japan, were examined. Patients who had blood transfusions during the perioperative period were excluded from the study to avoid extra-loading of bilirubin from a degradation of hemoglobin. Patients who had preoperative liver or renal dysfunction or postoperative complications were also excluded. The mean age of the 41 male and 55 female patients was 55.1 ± 16.9 years; the range was 15 to 86 years. Table I lists the diagnoses and number of patients studied. The diagnoses were made on the basis of operative findings and pathological examination. There was no statistical difference in the value of bilirubin and BOM between benign ($n = 56$) and malignant ($n = 38$) diseases.

The patients were divided into minor and major surgery groups according to surgical procedures (Tab. II). The patients were first divided into two categories, abdominal and non-abdominal (superficial) operations. However, since the number of non-abdominal procedures was small, the former group were further divided into two subgroups, minor and major laparotomies. The former included simple surgeries with short operation time, whereas the latter complicated surgeries with long operation time. The non-abdominal surgeries and the minor laparotomies were combined and designated as the minor operation group and the major laparotomies as the major surgery group.

TABLE II Classification of the patients by surgical procedures

| Surgical procedure | Number of patients |
|---|---|
| Minor operation | **57** |
| Non-laparotomy | |
| Mastectomy | 6 |
| Thyroidectomy | 6 |
| Inguinal herniorrhaphy | 3 |
| Other non-laparotomy | 5 |
| Minor laparotomy | |
| Cholecystectomy | 22 |
| Local resection of gastrointestinal tract | 4 |
| Other minor laparotomy | 11 |
| Major operation | **39** |
| Gastrectomy | 17 |
| Colorectal resection | 11 |
| Distal pancreatectomy | 3 |
| Pancreaticoduodenectomy | 2 |
| Subtotal esophagectomy | 1 |
| Other major laparotomy | 5 |
| Total | **96** |

Features of the patients in the two groups are listed in Table III. The minor surgery patients consisted of 18 males and 39 females ranging from 15 to 86 years of age, with an average of 51.1 ± 16.8 years. The major surgery patients consisted of 23 males and 16 females ranging from 20 to 80 years of age, with an average of

TABLE III Comparison between the two surgical categories

| Group | $n$ | Age (yr)† | Sex (m/f) | Operation time (min)† | Blood loss (ml)† |
|---|---|---|---|---|---|
| Minor surgery | 57 | 51.1 (16.8) | **18/39** | 160 (91) | 113 (130) |
| Major surgery | 39 | 61.0 (15.5) | **23/16*** | 272 (111)* | 550 (464)* |
| Total | 96 | 55.1 (16.9) | **41/55** | 205 (114) | 291 (377) |

$n$: number of the patients; m: male; f: female.
† values are mean (s.d.); * $p < 0.01$ *vs* minor surgery.

61.0 ± 15.5 years. Although age was not a significant factor, there was a statistical difference in sex between the two groups ($p < 0.01$). Mean operation time for major surgery was 272 ± 111 min and its mean blood loss volume was 550 ± 464 ml. Both data values are significantly greater than those of the minor surgery group with 160 ± 91 min mean operation time and 113 ± 130 ml mean blood loss volume ($p < 0.01$).

### Sampling and ELISA Procedure

Urine samples of 10 ml were collected from each patient in the morning before surgery and on days 1, 3, 5, 7, 10 and 14 after surgery. They were stored at −20°C in the dark until use. The concentration of BOM was measured using ELISA with anti-bilirubin monoclonal antibody as described previously by Shimizu [10] and Izumi [11]. Briefly, 300 μl of urine and 300 μl of saline were mixed well. Forty microlitres of this mixture was incubated with 100 μl of antibody solution for 20 min at 37°C to reach equilibrium. One hundred microlitres of the mixture was transferred to immunoplates (Nunc, Roskilde, Denmark) that had been coated with 100 μl/well (10 μg protein/well) of bilirubin-bovine serum albumin (BSA) and then blocked with 100 μl of gelatin-fortified phosphate-buffered saline (PBS). Free remaining antibody in the mixture was allowed to bind to bilirubin-BSA on immunoplates. After a 30 min incubation at 37°C, the immunoplates were washed 5 times, and 100 μl of the substrate solution was added. A green color developed as a result of peroxidase reaction. Thirty minutes later, absorbance was measured at 415 nm by ELISA analyzer BEP III (Hechist, Tokyo, Japan). A calibration curve was plotted as the ratio of $B/B_0$ ($B$: mean absorbance for each standard, $B_0$: mean absorbance of the control) against the bilirubin concentrations expressed as log ($i$) ($i$: bilirubin concentration). The concentrations of BOM were determined by linear regression analysis.

### Laboratory Examination

Serum total bilirubin concentration was analyzed by autoanalyzer (Hitachi 736-40 Automatic Analyzer, Hitachi Co. Ltd, Tokyo, Japan) during the same period described above.

### Statistical Analysis

Data were analyzed using the StatView J-4.5 software program (Abacus Concepts, Berkeley, California, USA). Results are expressed in terms of mean. Wilcoxon signed-ranks test was used to assess the differences between pre- and postoperative means. Regression analysis and Spearman's rank correlation coefficients were used to determine the relationships between different variables, and chi square test or the nonparametric Mann-Whitney U test was used to assess the difference between minor and major surgery groups. $P < 0.05$ was considered significant.

### RESULTS

### Changes in Serum Bilirubin and Urinary BOM in all Patients

The mean ± SEM concentrations of serum bilirubin and BOM in urine of all patients before and after surgery are depicted in Figure 1. Before surgery, serum bilirubin level was 0.71 ± 0.03 mg/dl. It increased and reached a peak of 0.96 ± 0.04 mg/dl on day 1 and decreased to the preoperative level on day 5. On day 14, the level of serum bilirubin was lower than the preoperative level ($p < 0.01$). The BOM level in urine was 0.75 ± 0.05 µmol/g creatinine before surgery. It increases slowly and reached a peak level of 0.97 ± 0.10 µmol/g creatinine on day 7 and then decreased gradually to the preoperative level on day 14. Peak serum bilirubin level on day 1 was significantly higher than the preoperative level ($p < 0.01$), but the increase of BOM in urine on day 7 was not statistically significant ($p = 0.11$).

### Comparison between Minor and Major Surgical Cases

To examine whether the levels of urinary BOM would be influenced by the degree of surgical stress and whether the increase of BOM would be significant in any certain population, we divided the whole group of patients into two groups as mentioned above (Tabs. II and III). The mean ± SEM concentration of BOM in urine in each group was shown in Figure 2. While the urinary BOM level was essentially constant in the minor surgery group, it increased from day 1 in the major surgery group and reached a maximum level of 1.30 ± 0.20 µmol/g creatinine on day 7, returning to the preoperative level on day 14. The increase on day 7 was statistically significant when compared with the preoperative value ($p = 0.048$). In a comparison between the two groups, the BOM concentration was significantly higher in the major surgery group on days 1, 5 and 7 ($p < 0.01$). In contrast,

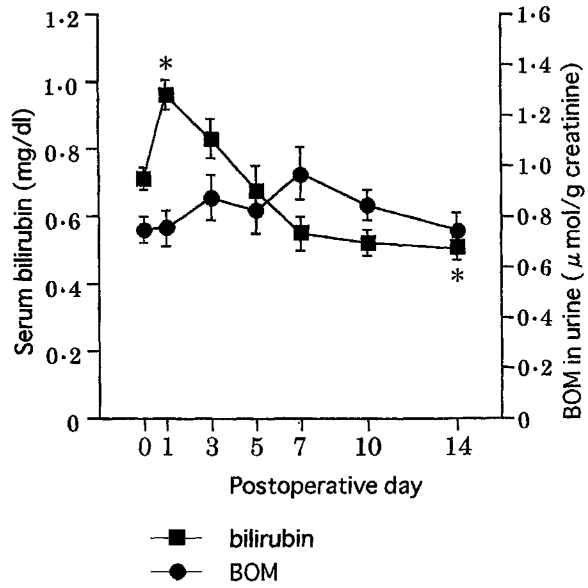

FIGURE 1 The level of serum bilirubin and urinary BOM in all patients. ■ serum bilirubin; ● urinary BOM. Results are mean ± SEM. *$p < 0.01$ *versus* day 0, indicating "before surgery".

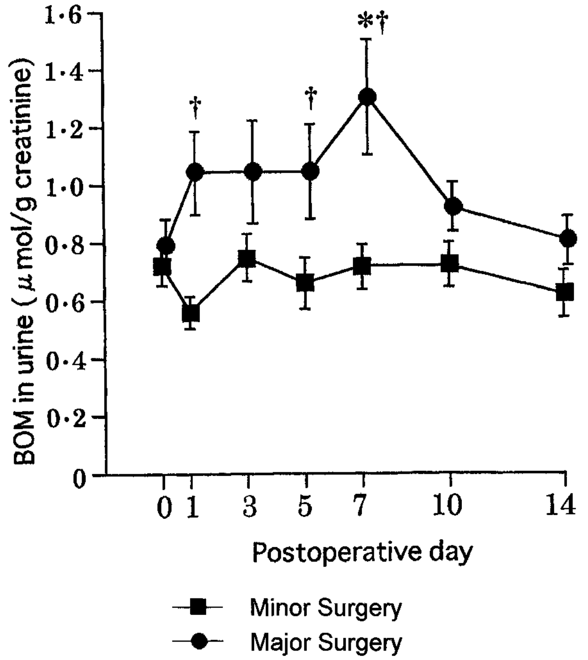

FIGURE 2 The level of urinary BOM in minor and major surgery patients. ■ minor surgery; ● major surgery. Results are mean ± SEM. *$p < 0.05$ *versus* day 0, indicating "before surgery". †$p < 0.05$ *versus* minor surgery group.

the levels of serum bilirubin showed no difference between the two groups, and the increase observed on day 1 for the total patients (Fig. 1) was also demonstrated in each group.

### Correlation of Serum Bilirubin and Urinary BOM with Operation Time and Blood Loss

In Figure 3, the correlation between operation time and the maximum level of serum bilirubin after surgery is shown. There was no significant correlation between these two factors ($p = 0.53$). However, the correlation between operation time and the maximum level of BOM in urine after surgery, shown in Figure 4, was significant on a linear curve ($y = 0.004{*}10^{-4}x + 0.563$, $r = 0.42$, $p < 0.01$). According to the analysis of blood loss, a significant correlation was similarly shown with BOM only ($y = 0.001{*}10^{-4}x + 0.989$, $r = 0.43$, $p < 0.01$), and no linear correlation was observed between serum bilirubin and blood loss ($p = 0.28$).

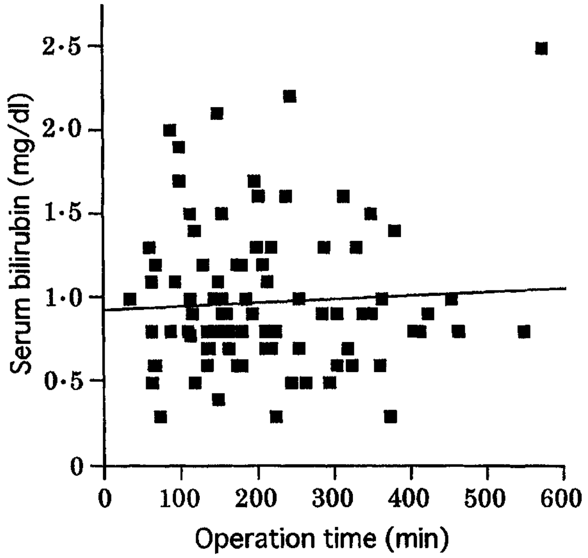

FIGURE 3 Correlation between operation time and maximum concentration of serum bilirubin. ($y = 2.48{*}10^{-4}x + 0.92$, $r = 0.07$, $p = 0.53$).

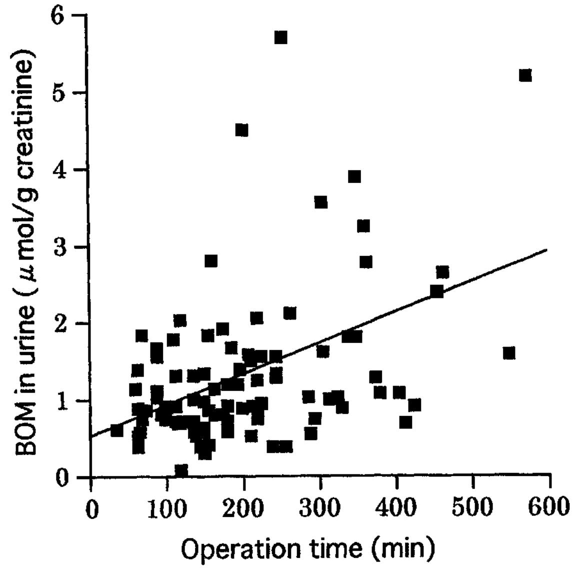

FIGURE 4 Correlation between operation time and maximum concentration of urinary BOM. ($y = 0.004{*}10^{-4}x + 0.56$, $r = 0.42$, $p < 0.01$).

### DISCUSSION

Bilirubin is synthesized from heme by a rate-limiting enzyme, heme oxygenase. It has been known as one of the "heat shock proteins," being dramatically induced in response to a variety of stress-associated agents including ultraviolet *A* (320–380 nm) irradiation, hydrogen peroxide ($H_2O_2$), and heavy metals as well as tumor promoters [12–14]. Endotoxin, tumor necrosis factor (TNF), interleukin 1 (IL-1) and interleukin 6 (IL-6) have also been recognized as stimulants [15–18]. It is of interest that all of these stimulating reagents lead to the production of reactive oxygen species [13], which means the induction of heme oxygenase is a general response to a variety of oxidant stresses in many mammalian cells [19].

The fact that bilirubin, the end-product of this activated enzyme, is proved to be a powerful antioxidant seems reasonable with respect to the fact that bilirubin is formed actively in stress conditions in order to neutralize the generated oxidants. In order to evaluate the antioxidant activity of bilirubin *in vivo*, Yamaguchi *et al.* established an ELISA system to measure BOM [7]. They first detected substances other than bilirubin that interacted with anti-bilirubin monoclonal antibody. They purified these from human urine and determined the structures of two compounds [7]. In contrast to bilirubin, which is a tetrapyrrole, BOM consist of three pyrrole rings and are thought to be produced when bilirubin molecules are destroyed oxidatively at the site of the pyrrole rings *A* or *D* [7]. These oxidative metabolites of bilirubin were subsequently proved to increase markedly by an endotoxin injection in the urine of rats in accordance with the elevation of liver heme oxygenase [6]. These results confirmed that bilirubin was oxidized in rats, acting as a physiological antioxidant and that the measurement of BOM in urine reflected the oxidant activity of bilirubin.

Our data described herein is summarized as follows: (1) Serum bilirubin significantly increased shortly after surgery regardless of the severity of surgery. (2) The concentration of BOM in urine elevated only after major surgery and the BOM levels in the major surgery group were higher than those in the minor surgery group. (3) The degree of increase in urinary BOM, but not the level of serum bilirubin, was statistically correlated with the length of operation time and the volume of blood loss.

The elevation of serum bilirubin reached a maximum level as early as the next day after surgery. The mRNA of heme oxygenase has been reported to be induced in the early phase of reaction against stress, being highest around 14 hours in fibroblasts after irradiation [20], and around 5 hours in rats after endotoxin injection [6]. The activity of the enzyme produced in rats was also demonstrated to be high as early as 6 hours after endotoxin injection [6]. The elevation of bilirubin was detected in the similar range of time. This confirms the hypothesis that bilirubin is actively synthesized by the enzyme. The measurements of mRNA or enzyme activity were not carried out in the present study since this would require samples to be collected from the liver or spleen of the patients repeatedly after surgery. Animal models are needed to obtain further direct confirmation of our results. In addition, findings that the increase of bilirubin occurred both in minor and major surgeries, and that there was a lack of correlation between the maximal level of serum bilirubin and the operation time or blood loss, suggested that bilirubin would be produced readily by minimal operative procedures. This phenomenon seems similar to the reaction of heme oxygenase mRNA in that levels close to maximum mRNA levels are reached at the low end of the dose range in all patients with radiation to human skin fibroblasts [19]. Induction of heme oxygenase to produce bilirubin seems to have a low threshold related to oxidative stress.

The reason why bilirubin returned to a level lower than the preoperative level 14 days after surgery is unclear (Fig. 1). One possibility is an overconsumption of bilirubin. It is reported that cigarette smoke, which is also known to be an oxidant, significantly lowers the plasma level of bilirubin in humans [21]. If the generated oxygen radicals exceed the production of bilirubin, the level of serum bilirubin decreases to a lower concentration. The contribution of difference in sex to our conclusion is uncertain.

The increase of BOM in urine after surgery confirmed that bilirubin acted as antioxidant during surgery in humans, similarly to that shown *in vitro* [5] or *in vivo* using rats treated with endotoxin [6]. The correlation of operation time and blood loss with BOM but not with bilirubin suggested that the amount of bilirubin to be used as antioxidant could be determined by the amount of generated reactive oxygens, whereas bilirubin itself was quickly induced by the smallest surgical stress as a spontaneous physiological response to potential oxidants. Because the increased release of superoxide anion from monocytes or neutrophils was reported in more invasive surgeries [22], BOM could be produced in accordance with the severity of surgical injury. When loaded with more severe surgical stress, more bilirubin was consumed to scavenge more oxidants. As a result, more BOM was excreted into urine after long and major surgeries. Urinary BOM, but not serum bilirubin, could be a good marker for evaluating the severity of surgical stress.

Bilirubin has been regarded as a cytotoxic, lipid-soluble waste. As discussed above, however, it plays an important role in patients with surgical stress. When incorporated into the model of a cell membrane, it has been reported to eliminate peroxyl radicals for lipid peroxidation to an extent beyond $\alpha$-tocopherol, which has been regarded as the best antioxidant of lipid peroxidation [5]. Moreover, albumin-bound bilirubin, shown to have antioxidant properties, could appear in inflammatory exudate across the vascular wall into the sites of increased production of oxygen radicals, since the binding of bilirubin to albumin distributes the pigment throughout the entire circulation and extravascular space [23]. There are lots of oxidant scavengers other than bilirubin, but their interactions are not clearly understood. Further studies are necessary to elucidate the mechanism of induction of bilirubin and the metabolism of BOM, and to investigate the other disorders related to the antioxidant activity of bilirubin.

### References

[1] Cowger, M. L., Igo, R. P. and Labbe, R. F. (1965). The mechanism of bilirubin toxicity studied with purified respiratory enzyme and tissue culture systems. *Biochemistry*, **4**, 2763-2770.

[2] Fan, S. T., Lo, C. M., Lai, E. C. S., Yu, W. C. and Wong, J. (1994). T lymphocyte function in patients with malignant biliary obstruction. *Journal of Gastroenterology and Hepatology*, **9**, 391-395.

[3] Ding, J. W., Andersson, R., Soltesz, V., Willen, R. and Bengmark, S. (1994). Obstructive jaundice impairs reticuloendothelial function and promotes bacterial translocation in the rat. *Journal of Surgical Research*, **57**, 238-245.

[4] Ding, J. W., Andersson, R., Norgren, L., Stenram, U. and Bengmark, S. (1992). The influence of biliary obstruction and sepsis on reticuloendothelial function in rats. *European Journal of Surgery*, **158**, 157-164.

[5] Stocker, R., Yamamoto, Y., McDonagh, A. F., Glazer, A. N. and Ames, B. N. (1987). Bilirubin is an antioxidant of possible physiological importance. *Science*, **235**, 1043-1046.

[6] Yamaguchi, T., Horio, F., Hashizume, T., Tanaka, M., Ikeda, S., Kakinuma, A. and Nakajima, H. (1995). Bilirubin is oxidized in rats treated with endotoxin and acts as a physiological antioxidant synergistically with ascorbic acid *in vivo*. *Biochemical and Biophysical Research Communications*, **214**, 11-19.

[7] Yamaguchi, T., Shioji, I., Sugimoto, A., Komoda, Y. and Nakajima, H. (1994). Chemical structure of a new family of bile pigments from human urine. *Journal of Biochemistry*, **116**, 298-303.

[8] Imamura, Y., Yokoyama, T., Murakami, Y., Hiyama, E., Takesue, Y., Kodama, T. and Matsuura, Y. (1995). Mac-1 expression and superoxide generation of the peripheral polymorphonuclear leukocyte following gastrectomy and esophagectomy. *Hiroshima Journal of Medical Sciences*, **44**, 39-46.

[9] Anderson, B. O., Poggetti, R. S., Shanley, P. F., Bensard, D. D., Pitman, J. M., Nelson, D. W., Whitman, G. J. R., Banerjee, A. and Harken, A. H. (1991). Primed neutrophils injure rat lung through a platelet-activating factor-dependent mechanism. *Journal of Surgical Research*, **50**, 510-514.

[10] Shimizu, S., Izumi, Y., Yamazaki, M., Shimizu, K., Yamaguchi, T. and Nakajima, H. (1988). Anti-bilirubin monoclonal antibody. I. Preparation and properties of monoclonal antibodies to covalently coupled bilirubin-albumin. *Biochimica et Biophysica Acta*, **967**, 255-260.

[11] Izumi, Y., Yamazaki, M., Shimizu, S., Shimizu, K., Yamaguchi, T. and Nakajima, H. (1988). Anti-bilirubin monoclonal antibody. II. Enzyme-linked immunosorbent assay for bilirubin fractions by combination of two monoclonal antibodies. *Biochimica et Biophysica Acta*, **967**, 261-266.

[12] Tomaro, M. L., Frydman, J. and Frydman, R. B. (1991). Heme oxygenase induction by $CoCl_2$, Co-protoporphyrin IX, phenylhydrazine, and diamide: evidence for oxidative stress involvement. *Archives of Biochemistry and Biophysics*, **286**, 610-617.

[13] Keyse, S. M. and Tyrrell, R. M. (1987). Both near ultraviolet radiation and the oxidizing agent hydrogen peroxide induce a 32-kDa stress protein in normal human skin fibroblasts. *Journal of Biological Chemistry*, **262**, 14821-14825.

[14] Keyse, S. M. and Tyrrell, R. M. (1989). Heme oxygenase is the major 32-kDa stress protein induced in human skin fibroblasts by UVA radiation, hydrogen peroxide, and sodium arsenite. *Proceedings of National Academy of Sciences of United States of America*, **86**, 99-103.

[15] Bissell, D. M. and Hammaker, L. E. (1976). Cytochrome P-450 heme and the regulation of hepatic heme oxygenase activity. *Archives of Biochemistry and Biophysics*, **176**, 91-102.

[16] Rizzardini, M., Carelli, M., Babello Porras, M. R. and Cantoni, L. (1994). Mechanisms of endotoxin-induced haem oxygenase mRNA accumulation in mouse liver: synergism by glutathione depletion and protection by N-acetylcysteine. *Biochemical Journal*, **304**, 477-483.

[17] Rizzardini, M., Terao, M., Falciani, F. and Cantoni, L. (1993). Cytokine induction of haem oxygenase mRNA in mouse liver; interleukin 1 transcriptionally activates the haem oxygenase gene. *Biochemical Journal*, **290**, 343–347.
[18] Mitani, K., Fujita, H., Kappas, A. and Sassa, S. (1992). Heme oxygenase is a positive acute-phase reactant in human Hep3B hepatoma cells. *Blood*, **79**, 1255–1259.
[19] Applegate, L. A., Luscher, P. and Tyrrell, R. M. (1991). Induction of heme oxygenase: a general response to oxidant stress in cultured mammalian cells. *Cancer Research*, **51**, 974–978.
[20] Vile, G. F. and Tyrrell, R. M. (1993). Oxidative stress resulting from ultraviolet *A* irradiation of human skin fibroblasts leads to a heme oxygenase-dependent increase in ferritin. *Journal of Biological Chemistry*, **268**, 14678–14681.
[21] Chan-Yeung, M., Ferreira, P., Frohlich, J., Schulzer, M. and Tan, F. (1981). The effects of age, smoking, and alcohol on routine laboratory tests. *American Journal of Clinical Pathology*, **75**, 320–326.
[22] Redmond, H. P., Watson, R. W. G., Houghton, T., Condron, C., Watson, R. G. K. and Bouchier-Hayes, D. (1994). Immune function in patients undergoing open *vs.* laparoscopic cholecystectomy. *Archives of Surgery*, **129**, 1240–1246.
[23] Stocker, R., Glazer, A. N. and Ames, B. N. (1987). Antioxidant activity of albumin-bound bilirubin. *Proceedings of National Academy of Sciences of United States of America*, **84**, 5918–5922.

### COMMENTS

Reactive oxidative species (ROS) are known to cause damage to cells/tissues, in a number of conditions, such as sepsis and surgical stress. A number of products, including cytokines (TNF$\alpha$ and IL-6 for example), are known to induce the production of these products. Parameters that can directly or indirectly reflect these oxidative events, have important bearings to assess the degree of tissue damage and injuries. The study by Kozaki and colleagues demonstrates that bilirubin oxidative metabolites, correlates with the type of surgical procedure, operating time and blood loss. These observations, together with those previously reported that the bilirubin oxidative metabolites are indicators of oxidative damage in animal studies, suggest a possible beneficial nature to monitor these products in clinical practice. Furthermore, these oxidative products can be measured in urine with a well-established technique, making the test an attractive and non-invasive option. Studies on other type of diseases, such as sepsis may be important to establish this context.

Dr. Wen G. Jiang, M.D.
Senior Lecturer
University Department of Surgery
University of Wales College of Medicine
Cardiff, UK

